# Quantitative outcome of registration methods for correcting cardiac drift in cardiac PET/CT imaging

**DOI:** 10.1120/jacmp.v17i2.5806

**Published:** 2016-03-08

**Authors:** Jonathon A. Nye, Dana Tudorascu, Fabio Esteves, John R. Votaw

**Affiliations:** ^1^ Department of Radiology and Imaging Sciences Emory University Atlanta GA USA

**Keywords:** PET/CT, attenuation correction, image registration, artifacts, cardiac imaging

## Abstract

Myocardial perfusion studies suffer from artifacts caused by misalignment of the transmission and emission data due to the influences of voluntary and involuntary patient motion. Regardless of G68e or respiratory‐averaged CT based attenuation correction and good patient cooperation, approximately 21% of perfusion studies exhibit artifacts arising from misalignment that cannot be corrected by manipulating the attenuation acquisition protocol. This misalignment, termed cardiac drift, is caused by slow‐moving abdominal cavity contents that reposition the heart in the thorax and appear as myocardial uptake overlying the left CT lung in fused PET/CT images. This study evaluates three postimaging registration techniques to correct PET/CT misalignment by altering the transmission map to match myocardial uptake. Simulated misalignment studies were performed with a cardiac torso phantom filled with [F18]FDG at 10:1 myocardium/background. An air‐filled saline bag affixed to the medial left lung surface served as a distensible lung. An initial CT acquisition was followed by successive PET acquisitions consisting of small displacements of the cardiac insert into the left lung. Phantom transmission scans were aligned to the myocardial uptake in the emission scans by applying 1) full rigid‐body translations and rotations, 2) rigid‐body restricted to medial / lateral and superior / inferior translation, or 3) an emission‐driven method that adds myocardial tissue to the transmission scan. These methods were also applied to 10 low‐likelihood coronary artery disease (CAD) patients showing signs of cardiac drift. Full rigid‐body registration showed significant over‐correction (p<0.004) of activity concentrations in the artifact areas of the phantom data due the relocation of highly attenuating structures (i.e., spine). Inaccurate regional activity distributions were also observed as streaks extending from the spine and these results were replicated in the patient population. There was no significant difference between the true phantom activity concentration after correction with the emission‐driven method. Misalignment corrected with the rigid‐body registration results in an increase in activity concentration but fails to accurately recover the true concentration. These data suggest that a nonlinear image registration approach such as an emission‐driven method results in a more uniform activity distribution throughout the myocardium, and is more appropriate for addressing the cardiac drift misalignment problem.

PACS number(s): 87.57.uk, 87.57.Q, 87.57.nj, 87.57.C, 87.19.Hh

## I. INTRODUCTION

Cardiac imaging with positron emission tomography (PET) can provide quantitative information on myocardial perfusion and metabolism.^1^, ^2^ A requisite for recovering absolute and regional activity concentrations is proper registration between the transmission and emission acquisitions prior to reconstruction.^(3)^ Accurate registration becomes compromised in the presence of patient motion where the attenuating structures estimated by the transmission study do not correspond to the myocardial tissue uptake distribution in the emission study. The registration problem is most noticeable along sharp tissue‐air interfaces such as the left lung‐left ventricular wall and observed as myocardial tissue uptake overlaying the transmission lung field in the fused images. In this case, myocardial uptake present in the transmission lung field is assigned lower attenuation values consistent with lung tissue resulting in emission images with regions of artificially low myocardial uptake.

Registration artifacts that arise from misalignment of cardiac tissue boundaries are caused by voluntary patient motion, respiratory motion, contractile cardiac motion, and drift of the thoracic cavity contents. Voluntary patient motion commonly occurs in response to patient discomfort and rigid‐body transformations applied to the transmission image to match regions of myocardial uptake have resulted in the recovery of artificially low count regions.^(4)^ In dedicated PET systems, the long transmission times required when using rotating G68e transmission sources results in nearly equivalent respiratory and cardiac temporal resolution. With the introduction of computed tomography (CT) for attenuation correction, the acquisition speed of helical CT fails to account for the time averaging observed in the emission acquisition.^(5)^ As a result, the reported frequency of registration errors between the transmission and emission sequences increased from 21% in dedicated PET system^(6)^ to 71% in PET/CT hybrid systems.^5^, ^7^ Studies have focused on altering the CT transmission acquisition to incorporate the effects of respiratory and cardiac motion, including averaging cine acquisitions collected over one or more breath cycles^8^, ^9^ and low‐pitch CT protocols.^(7)^ The use of cardiac attenuation correction specific protocols have resulted in a substantial reduction in misalignment, which is now comparable to dedicated PET systems, as observed by Loghin and colleagues.^(6)^

Approximately one‐quarter of the PET/CT cardiac studies exhibit misalignment due to the slow, continuous movement of the thoracic cavity contents,^6^, ^7^ referred to as cardiac drift in this work. Further reduction in misalignment requires a postprocessing software solution, as the nature of the cardiac drift problem cannot be addressed by altering the transmission or emission acquisition protocols. Two groups showed that rigid‐body transformation of the transmission map to match areas of myocardial uptake caused a significant increase in activity in regions of misregistration.^5^, ^6^, ^10^ However, the proposed corrections lacked quantitative analysis to show whether the recovered activity concentrations were representative of the true activity concentration distribution. The purpose of this work was to investigate the quantitative accuracy of rigid‐body image registration of PET and CT by simulating the cardiac drift problem in a phantom and applying these approaches to a normal patient cohort exhibiting cardiac drift.

## II. MATERIALS AND METHODS

### A. Scanner

Phantom studies were acquired on a GE Discovery LS (General Electric Medical Systems, Milwaukee, WI), comprised of a full 4.0×8.1×30 mm bismuth germanate (BGO) block detector ring of 150 mm field‐of‐view operating in the 2D mode.^(11)^ Patient studies were acquired on a GE Discovery ST, which is constructed with a full 6.0×6.0×30 mm BGO block detector ring of 157 mm FOV also operating in the 2D mode.^(12)^ The CT employed on these scanners is a GE LightSpeed 16‐slice detector array with a tube current range of 10–140 mA, peak kilovoltage range of 80–140 kVp, and maximum gantry rotation frequency of 3 Hz.

### B. Phantom studies

**Figure 1:**
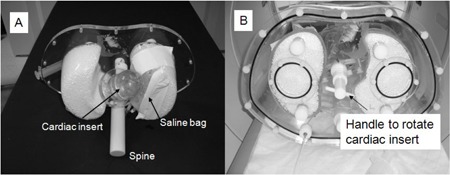
Photograph of the phantom setup with arrows showing the placement of the air‐filled saline bag, cardiac insert and spine (a) and handle for rotating the cardiac insert (b). In addition, the phantom contains two Styrofoam — water filled lungs, liver (not shown), and body cavity.

Simulations of cardiac drift between the transmission and emission sequences were performed with an anthropomorphic torso phantom (Data Spectrum Inc., Hillsborough, NC) using a cardiac insert without defects. Lung inserts were filled with Styrofoam beads and back‐filled with water. The sharp left ventricular/left lung interface observed in clinical studies was reproduced by affixing a 250 mL air‐filled saline bag to the medial surface of the left lung. Access to the saline bag from outside the phantom was accomplished by a bored cap screw threaded with a tube allowing for adjustment of the air volume such that it contoured the cardiac insert. The cardiac insert was secured to the phantom with a modified screw that included an extension with a handle permitting rotation in situ of the insert towards and away from the distensible saline bag. The cardiac insert was joined to the torso phantom at its base permitting rotations that appear as movement of the left ventricle apex away from the medial plane and into the left lung (Fig. 1).

The myocardial cavity was filled with [^18^F]‐fluoro‐2‐deoxy‐glucose at a concentration of 10:1 relative to the background regions (e.g., left and right lung, liver, body, blood pool) with the phantom oriented in the supine position on the scanner bed. Cardiac drift was simulated by first acquiring an early CT with the cardiac insert positioned away from the left lung. The cardiac insert was then rotated into the air‐filled saline bag and an emission study was collected consisting of a 10‐minute acquisition. A late CT exam was acquired following the emission with the cardiac insert in the rotated position. Four scans were performed in total, beginning with the cardiac insert in the out position and applying four graduated rotations of the cardiac insert into the distensible saline bag. Three reconstructions were performed: 1) the early CT with the emission, which mimics the cardiac drift observed in patient studies and results in an artificial defect, 2) the early CT registered to the emission using one of the three methods discussed below, and 3) the late CT with the emission, which provides a true representation of the attenuating structures and hence the true reconstructed activity concentration in the presence of the simulated cardiac drift. Emission data were corrected for attenuation, scatter, and randoms and reconstructed using a 2D ordered‐subsets expectation maximization (OS‐EM) algorithm.

### C. Registration methods

The amount of cardiac drift was assessed using a variation of the method presented by Loghin et al.,^(6)^ where cardiac uptake from the emission acquisition overlaying the left lung field of the early CT transmission was identified.^(7)^ Briefly, the method applies a simple threshold technique to separate the pixels into regions of myocardial uptake and attenuating tissue. Myocardial uptake in the emission exam was identified by finding the maximum pixel value in the cardiac volume and taking 50% of that value as a lower threshold. Emission pixel values falling between the lower threshold and maximum myocardial pixel were set to a value of one with all other pixels set to zero. Similarly, a binary map of attenuating structures in the transmission image was created by setting Hounsfield values greater than lung tissue (−450 HU) to a value of one and all other pixels to zero. The emission and transmission binary representations were then overlaid using the native scanner registration. A watershed algorithm applied to the left lung of the transmission image identified shared pixels between the binary transmission lung field and binary myocardial uptake image. The number of emission pixels overlaying the transmission lung field yielded the magnitude of misalignment expressed in units of volume (mL).

Three registration methods were employed to recover the activity distribution from regions of reduced counts resulting from the simulated cardiac drift. Each method consisted of manipulations applied to the early CT image, prior to rotation of the cardiac insert into the distensible air‐filled saline bag. This was analogous to acquiring a single CT prior to administration of a PET perfusion agent. The first registration method consisted of a full (3 translation, 3 rotation) rigid‐body transformation applied to the CT transmission image to match areas of cardiac tissue with that of myocardial uptake measured in the emission image. The second registration method was also a rigid‐body registration but limited to translation of the CT transmission image in the medial / lateral and superior / inferior direction (labeled as medial‐axial) such that the entire myocardiac uptake region was placed in the mediastinal cavity of the phantom. This case attempts to replicate movements permissible on an imaging bed, which is restricted in the anterior / posterior direction. The third registration method was an emission‐driven approach that reassigns shared overlaying pixels in the early CT new Hounsfield values consistent with myocardial tissue.^(5)^ Therefore, pixel values previously designated as lung tissue in the early CT image were reassigned as tissue values in regions of overlaying myocardial uptake using the technique described above.

The late CT attenuation image represents the true reconstructed position of the attenuating structures following rotation of the cardiac insert. Therefore, emission data reconstructed with the late CT were used for comparison against the two rigid‐body and emission‐driven registration methods. Analysis of the registration techniques was performed by drawing regions of interest (ROI) over the lateral wall of the left‐ventricle insert in the reconstructed emission image using the late CT for attenuation correction. The same regions were then applied to the PET images reconstructed with each of the image registration methods. Percent recovery was calculated by dividing the ROI from the reconstructed emission data with the registered early CT data by the emission data reconstructed with the late CT data. Lastly absolute valued differences images, Difference Image=(emission data reconstructed with registered early CT AC)−(emission data reconstructed with late CT AC), were created to highlight areas of artificial uptake caused by erroneous registration of the PET and CT data.

### D. Patient studies

The methods performed in the phantom simulations were applied to 10 patients who had less than 5% likelihood of coronary artery disease (CAD) based on sequential Bayesian analysis of age, gender, and symptom classification.^(13)^ Also, these patients had a normal baseline ECG, no induced ECG changes after pharmacological stress, no major risk factors for CAD or a history of previous cardiac events. Rb‐82 myocardial perfusion exams were acquired on a GE DST scanner using a low‐pitch CT acquisition protocol to minimize the influences of respiratory and cardiac motion. The same registration techniques used for the phantom data were applied to the patient data to identify pixel indices corresponding to cardiac uptake present in the transmission lung field. The full rigid‐body transformation, medial‐axial rigid‐body transformation, and emission‐driven registration methods were applied to the CT image to best approximate the true myocardial tissue distribution and recover regions of reduced counts resulting from cardiac drift.

### E. Statistical analysis

A linear mixed effects model was applied to the phantom data to evaluate differences between the true myocardial concentration and the rigid‐body registration method. The linear mixed model offers a flexible way of modeling the registration method while accounting for the serial correlations in the repeated measures for each individual phantom scan. We fit a model with the differences between methods quantified separately for each measurement point in each phantom orientation.

Given the small sample size, patient data were analyzed using a Wilcoxon signed‐rank test, the nonparametric analogue to the matched pairs Student's *t*‐test, to avoid making the assumption that the patient data followed a normal probability distribution. To accommodate multiple comparisons, namely the three registration measures, a Bonferroni correction was applied adjusting the type‐I error threshold for significance to 0.83%. All statistical analyses were performed using SAS 9.1.3 (SAS Institute Inc., Cary, NC).

## III. RESULTS

**Figure 2:**
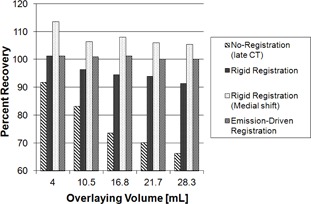
Plot of the percent recovery vs. overlaying volume measured in the phantom simulations for each of the three registration methods.

The recovery of the phantom myocardial activity, expressed as percent recovery of the true myocardial activity concentration, is shown in Fig. 2. Statistically significant differences were observed between the early CT reconstruction and the late CT reconstruction without any registration methods applied (p≤0.004). Statistically significant differences were also observed between the late CT reconstruction and each of the two rigid‐body registration techniques employing the full (p<0.002) and medial‐axial (p<0.002) rigid‐body methods. Similarly, the emission‐driven method resulted in significant increased activity compared to the late CT images (p<0.002).

**Figure 3:**
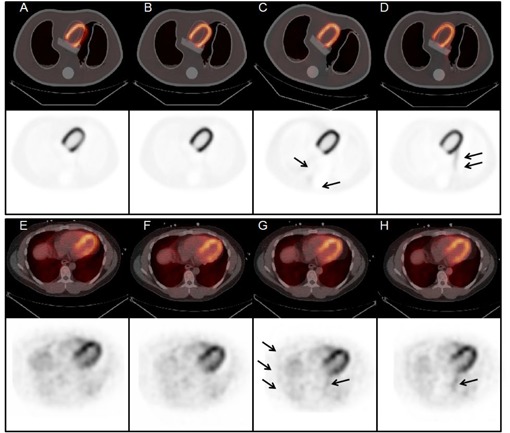
Fused and PET images of phantom simulations (top panel) and patient studies (bottom panel) reconstructed with the early CT ((a) and (e)), emission‐driven method ((b) and (f)), full rigid‐body ((c) and (g)), and medial‐axial rigid‐body ((d) and (h)) registrations. Arrows point to regions of artificially high uptake caused by misalignment of attenuating structures between the emission and transmission images.

**Figure 4:**
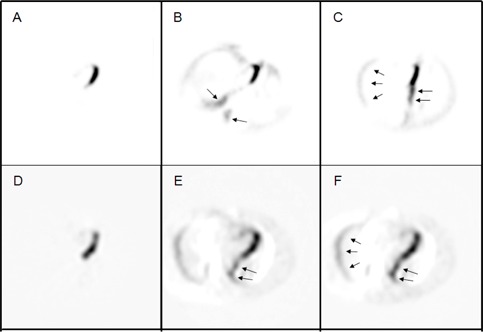
Difference images of reconstructed PET data of the phantom (top panel) and patient (bottom panel) images employing the emission‐driven ((a) and (d)), full rigid‐body ((b) and (e)), and medial‐axial rigid‐body ((c) and (f)) registration methods. Arrows point to areas of artificial update resulting from erroneous attenuation correction.

Simulations of large cardiac drift artifacts required similarly large rotations and medial shifts to match regions of myocardiac tissue uptake and attenuating tissue, respectively. Close inspection of the rigid‐body registration reconstructions reveals the presence of artifacts caused by the relocation of highly attenuating structures, particularly the spine. As the spine moves towards the left lung, an artificial increase in activity was observed along the border of the left lung and extending down from the left ventricular wall (Fig. 3). Regions of reduced counts were, therefore, present along lines of response previously corrected by the spine. Similarly, movement of the spine towards the right lung led to a reduction in activity in the left ventricular wall and an increase in activity along the anterior wall of the right lung. Regional activity distributions in the rigid‐body reconstructions are visibly altered by the large movements in the torso phantom CT image. Figure 4 shows difference images between each registration method and the early CT reconstruction. These images represent the absolute residual activity after applying each of the three registration methods and arrows highlight the artificially high uptake areas.

Misalignment of the myocardium in the patient data was consistent with the phantom simulation experiments of cardiac drift between the CT transmission and emission acquisitions. Application of the full and medial‐axial rigid‐body registration resulted in repositioning of the spine towards the left lung and artificial uptake along the lung boundaries following reconstruction (Fig. 4). The activity concentration along the lateral wall of the left ventricle increased significantly compared to the uncorrected (p<0.005). Application of the emission‐driven method resulted in increased activity concentration along the left ventricular lateral wall and was significant compared to the uncorrected images (p<0.005). No significant difference was observed between the medial‐axial rigid‐body registration and emission‐driven method, but the rigid‐body method trended higher (p=0.0195).

## IV. DISCUSSION

This study demonstrates that rigid‐body registration to correct PET/CT misalignment caused by cardiac drift does not result in accurate recovery of the true activity concentration. Our simulation studies in phantoms show that the error in myocardial activity concentration over the misaligned area may range between ±5% to 10%, depending on whether full or translation only rigid‐body registration is applied. The emission‐driven method performed better than rigid‐body registration at recovering the true activity concentration in the phantom simulations suggesting that a nonlinear registration approach is needed to address the cardiac drift problem.

The failure of the rigid‐body registration methods to correctly recover the myocardial activity distribution of the phantom is due to the relocation of highly attenuating structures. The most significant attenuating structure is the spine and when relocated creates streaks extending from the spinal location to the heart. Medial shift of the spinal column over‐corrects lines‐of‐response falling over the left ventricular wall leading to an artificial increase in activity concentration. The rigid‐body registration methods show improved activity concentration recovery in the myocardium but either over‐ or undercorrected the activity concentration depending on the position of the spine. Although the true activity distribution in the patient studies is not precisely known, the phantom studies successfully simulated the artificially high uptake along the lateral wall and demonstrated potential complications of applying rigid‐body registrations.

This work further suggests that a nonlinear registration approached is needed to estimate the appropriate transmission tissue distribution to accurately address the cardiac drift misalignment problem. The emission‐driven method is less technically difficult to implement compared to other deformable algorithms and groups have applied nonlinear warping algorithms to solve the PET/CT registration problem,^(14)^ though none have been extensively validated for cardiac perfusion imaging. The importance of accurate data correction in cardiac PET/CT not only affects interpretation of perfusion images but also more advanced analysis techniques, such as the calculation of absolute blood flow.^(15)^ Therefore centers performing cardiac PET/CT should be aware of pitfalls when applying image registration techniques and their affects on quantification.

## V. CONCLUSIONS

The use of rigid‐body registration methods to correct the cardiac drift misalignment problem improves the myocardial activity distribution but fails to quantitatively recover the true activity concentration. Relocation of highly attenuating structures such as the spinal column results in artificial increases in uptake and regional artifacts. The data presented in this work show that a nonlinear image registration approach is more appropriate to correct the cardiac drift problem. Careful consideration must be taken when aligning PET and CT to judge good alignment in all areas of the thorax.

## COPYRIGHT

This work is licensed under a Creative Commons Attribution 4.0 International License.
